# Providing Effective Mental Health Support for Oncology Health-Care Workers in the COVID-19 Era: Responding Quickly but Carefully

**DOI:** 10.1093/jncics/pkab031

**Published:** 2021-04-02

**Authors:** Ajay Major, Christopher J Recklitis, Sharon Bober

**Affiliations:** 1 Section of Hematology/Oncology, University of Chicago Medicine, Chicago, IL, USA; 2 Department of Pediatrics, Harvard Medical School, Boston, MA, USA; 3 Department of Psychiatry, Harvard Medical School, Boston, MA, USA

## Abstract

Oncology health-care workers (HCWs) are facing substantial stressors during the current coronavirus disease 2019 pandemic, resulting in a wide range of acute stress responses. To appropriately meet the growing mental health needs of HCWs, it is imperative to differentiate expectable stress responses from posttraumatic stress disorder and mental illness, because traditional mental health interventions may pathologize healthy stress reactions and risk retraumatizing HCWs under acute duress. Further, HCWs are experiencing protracted forms of acute stress as the pandemic continues, including moral injury, and require mental health interventions that are flexible and can adapt as the acuity of stressors changes. Previously developed frameworks to support people experiencing acute stress, such as Psychological First Aid, are particularly relevant for HCWs in the ongoing pandemic. Acute stress interventions like Psychological First Aid are guided by the Stress Continuum Model, which conceptualizes stress reactions on a continuum, from a zone of normal readiness and expectable consequences to a zone of more persistent and extreme reactions such as posttraumatic stress disorder and major depression. Key principles of the Stress Continuum Model include the expectation that emotional reactivity does not lead to psychiatric problems, that interventions need to be appropriately targeted to symptoms along the stress continuum, and that people will return to normal recovery. Various core actions to reduce acute stress include delivering practical assistance, reducing arousal, mobilizing support, and providing targeted collaborative services. This nonpathologizing approach offers a valuable framework for delivering both individual and organizational-level interventions during the coronavirus disease 2019 pandemic.

“I am not a hero. I am scared.” (1) These words of a resident physician, assigned to care for coronavirus disease 2019 (COVID-19) patients in New York City, are representative of the stress responses of frontline health-care workers (HCWs) thrust into this unprecedented and ongoing pandemic. Acute and chronic stress reactions, including anxiety, depression, and symptoms of posttraumatic stress disorder (PTSD), have been well-documented in HCWs after stressful events (2). However, the COVID-19 pandemic is unlike any other crisis to date for most HCWs in both scope and duration. HCWs may need to care for overwhelming numbers of critically ill patients while also confronting personal safety concerns (3). At the same time, HCWs may also face distressing ethical quandaries, ranging from scarce resource allocation to social distancing (4).

Fear of COVID-19 infection in cancer patients has radically changed the practice of oncology, with delays in diagnosis due to self-isolation and treatment interruptions in both the adjuvant and palliative settings (5). This fear is felt by both patients and oncology HCWs, with the recent COVID-NOW study by Banerjee et al. (6) demonstrating poor well-being in 42% and burnout in 34% of surveyed oncology HCWs in the United Kingdom. Another survey-based cross-sectional study in Canada found a greater than 50% prevalence of anxiety, depression, and hopelessness among oncologists (7). Oncologists and oncology HCWs may be at particular risk for stress during the pandemic as they witness their patients succumb to the infection and are unable to provide their patients with treatments due to widespread cessation of clinical trial accrual. As HCWs both on and off the front lines continue to report profound stress reactions, there are major calls for action (8,9), and institutions are urgently trying to respond to the mental health needs of their workers.

To appropriately meet the growing mental health needs of HCWs, it is imperative to differentiate expectable, acute stress responses from PTSD and mental illness. Viewing all HCW reactions to the pandemic through the lens of mental illness or trauma risks pathologizing what are normal reactions to highly abnormal circumstances. This can lead to confusing the emotionality and vulnerability of HCWs as psychological symptoms to be “managed” and potentially to discount the occupational and systemic drivers that underpin this stress.

At the same time, we must not fail to appreciate the intensity of HCWs’ stress and the profound effect it has on them. For example, the current pandemic has brought to bear a number of extreme stressors that most HCWs have never before encountered. “Moral injury,” a term used to capture the psychological consequences of perpetrating or witnessing immoral events on the battlefield, has recently been ascribed to the real-time ethical dilemmas that HCWs experience when they are unable to adequately care for their patients due to forces beyond their control (10). This particular type of stress injury has been anecdotally described, including by Dr Mark Lewis (11) in the *New England Journal of Medicine*, who describes the agonizing decisions oncologists face when they have to deintensify, delay, or cancel cancer treatments due to the pandemic. As HCWs face another wave of the surging pandemic, other examples of moral injury include the anguish from witnessing shortages of critical medical equipment, having to activate “crisis standards of care,” and guilt felt by being so physically and emotionally exhausted that one loses their capacity to empathize (12,13). Failing to appreciate the importance of moral injury risks underestimating HCWs’ stress and the need for responses that go beyond wellness promotion programs designed for individuals with more common everyday stressors. From a broader socio-cultural perspective, it is similarly important to acknowledge that for close to 12 months, HCWs have also faced stressors ranging from the politicization of mask-wearing to a steady stream of medical misinformation and disinformation that have been both undermining and culturally destabilizing during this time of international crisis (14).

Lessons from previous epidemics and precedent-setting work with first responders and military personnel can inform mental health interventions for HCWs given the current challenges of COVID-19. In the SARS epidemic of 2002-2003, 18% to 57% of HCWs experienced acute emotional distress during the outbreak, with worse distress associated with direct exposure to infected patients, quarantine, and interpersonal isolation (15,16). However, it is notable that the majority of distressed HCWs recovered after SARS subsided, with only a minority experiencing long-term mental health sequelae (17). As others have concluded, clinical interventions for reducing pandemic-related stress should turn towards models of fostering adaptation and resilience in psychologically healthy people rather than relying on clinical interventions primarily aimed at mental health problems (18). Consistent with this perspective, we also view observations of stress reactions in the current pandemic as congruent with the Stress Continuum Model (Figure 1), initially developed to describe stress reactions in military personnel (19).

**Figure 1.**
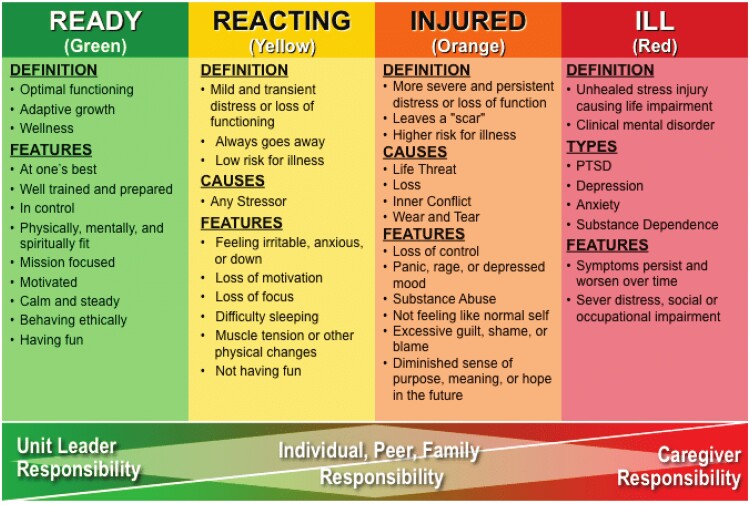
The Stress Continuum Model (19). This figure is courtesy of the United States Government, which does not have copyright protection. PTSD = posttraumatic stress disorder.

Describing 4 zones ranging from green to red, the Stress Continuum Model emphasizes the importance of viewing stress reactions on a continuum, from normal readiness and expectable consequences to more persistent and extreme imbalance in the red zone such as PTSD and major depression. The model underscores that stress reactivity may cycle between zones and emphasizes the importance of providing appropriate-level interventions to meet the needs of individuals where they are. Just as it is paramount to bring individuals experiencing “red zone” levels of distress back to a less severe zone, it is also important to recognize that mental health interventions aimed at red zone levels of distress may not be appropriate for individuals who are in the immediate phases of acute stress, such as described in the yellow and orange zones.

Interventions for managing acute stress have built on this continuum-based understanding and have been adapted for different circumstances and populations, the most well-known being Psychological First Aid (PFA) (20,21). Fundamental to PFA is the understanding that 1) although almost everyone has strong emotional reactions to acutely stressful circumstances, these reactions usually resolve within days or weeks; 2) that this emotional reactivity does not lead to permanent psychiatric damage; and 3) it is expected that individuals will achieve normal recovery (21). This nonpathologizing framework offers helpful tools that could also be adapted for HCWs in the time of an ongoing pandemic. In particular, PFA outlines several “core actions” or intervention principles that are essential for optimizing recovery in the face of acute stress, which include information on coping, reduction of physiological arousal, practical assistance, mobilization of support, and linkage with collaborative services (20,21). The PFA framework underscores the need to tailor these actions to the individual, either one-on-one or in a group setting (22). For example, at the individual level, a relevant example of reducing physiological arousal might include limiting excessive exposure to distressing stimuli such as news and social media and learning self-calming techniques such as breath awareness. An example of mobilizing support would be more enhanced virtual contact with friends and family. There are also implications of the PFA framework at the organizational level, such as providing HCWs with practical resources and adequate personal protective equipment as well as low- or no-cost access to stress reduction apps and online programs. Hospitals can also offer greater opportunity for physician peer support, such as building in time for team meetings, brief huddles, or establishing ongoing peer support groups. Peer support groups have been shown to be particularly valuable because physicians may be reluctant to accept more general counseling support (23). These core actions and strategies are applicable to HCWs and patients across oncologic practices. Table 1 offers specific examples of various individual- and systems-level interventions that can be used to support HCWs under acute stress (in the yellow and orange zones of the Stress Continuum Model) based on these acute stress intervention frameworks, including PFA.

**Table 1.** Examples of how existing intervention models can be adapted to support HCWs experiencing acute stress during the COVID-19 pandemic^a^

Intervention model components	Individual-level interventions	Systems- or organizational-level interventions
Engagement and orientation	Supervisors acknowledge HCWs’ current work stress and inquire about immediate concerns; emphasize listening to concerns, normalizing stress reactions, and avoiding pathologizing language and assumptions about “trauma” or “symptoms” Provide HCWs easy access to information and resources to manage stress (flyers, online resources, etc)	Hospital-wide messaging that acknowledges and validates acute stress reactions as normal and transient Create and maintain consistent sources of communication regarding changes in operations during COVID-19 (e-mails, webinars, etc) Create and maintain sources of information about practical resources, coping to reduce distress, and promotion of adaptive functioning
Practical assistance	Provide practical support to address concerns, such as temporary housing, childcare resources, parking and transportation resources Provide support staff with time for meals and breaks during shifts Provide professional resources to support HCW competence and safety in their professional roles, including PPE and in-service and continuing education related to COVID-19	Institutional support for flexible scheduling and work arrangements Provide additional parking, shuttle services, and on-site areas for sleep and rest Availability of no-cost professional well-being programs Support for resources such as hazard pay and enhanced time off Provide information on systems’ inventory of supplies and equipment (such as ventilators) and enact strategies to overcome staff and material shortages
Information gathering	Request information about HCWs’ needs directly and through supervisors and care teams Actively engage HCWs about suggestions to improve operations, personal safety, and well-being	Provide system-wide conduits for HCWs to share requests and suggestions anonymously Use system-wide forums to discuss identified needs and steps taken to address them
Provide education	Provide basic information through e-mail or flyers to HCWs about stress and coping, including normalizing strong reactions to stressful conditions; explaining the expectation that stress reactions subside with time after stressful conditions subside; recommendations for self-help interventions to enhance coping and reduce stress; availability of professional resources for stress reactions that impair functioning or that do not respond to self-care; provision of more detailed information about managing sleep, alcohol, and drugs, and concerns about children, elderly relatives, or helping a coworker who appears stressed	Tip sheets and educational resources readily available on shared business communication platforms Provide financial support for confidential peer-support groups and employee assistance programs
Recommend steps to reduce arousal	Support HCWs’ steps to reduce arousal by minimizing unnecessary discussions of stressful events and promoting “healthy avoidance” by limiting news and social media Encourage short (5-min) breaks in the work day when possible to destress Provide information and resources for relaxation exercises, paced-breathing, and mindfulness-based stress reduction	Reduction of expectations for nonessential work tasks Provide HCWs with low- or no-cost access to relaxation and stress reduction apps and online programs Availability of no-cost professional well-being programs
Recommend mobilization of support	Take steps to enhance social supports Build brief opportunities for virtual support and informal engagement with colleagues into the work week as part of team meetings or brief huddles Remind HCWs that spending time away from work with important others is helpful in managing stress	Institutional support for flexible scheduling and work arrangements Opportunities for peer support such as peer-led online support groups Encourage HCWs to take time for social support without negative repercussions, such as extending deadlines, decreasing nonessential work meetings, and pausing promotion clocks
Linkage with collaborative services	Provide clear and accessible links to available services and coping resources	Provide financial support for confidential peer-support groups and employee assistance programs Tip sheets and educational resources readily available on shared business communication platforms
Emphasize brevity and simplicity (BICEPS)	—	Develop information and resources that are clear and simple Breaks from work, adequate supplies, rest and sleep, nourishment, and clear communication may be more important than complex interventions or programs
Immediacy and proximity (BICEPS)	—	Interventions should be readily available in the HCWs’ environment and begin at the onset of stressful conditions

a Table adapted from Friedman (21). “—” indicates cell was intentionally left blank because BICEPS is an organizational-level intervention. HCW = health-care workers; PPE = personal protective equipment; BICEPS = Brevity Immediacy Centrality Expectancy Proximity and Simplicity.

It is clear that oncology HCWs need stress-coping resources that are evidence based and temporally appropriate. To achieve this, we recommend that health-care institutions provide mental health resources such as PFA that are specifically designed to support HCWs in acute stress conditions rather than traditional trauma debriefing modalities, which may in fact be deleterious. For example, asking individuals to recall details of highly stressful events, a technique called Critical Incident Stress Debriefing, can actually exacerbate distress when individuals are currently in the middle of an acute crisis. In fact, it has been shown that Critical Incident Stress Debriefing likely increases PTSD symptoms in acute stress situations (20). Similarly, defensive coping, often seen as a symptom of unresolved PTSD, may function as an effective coping strategy in an HCW reexposed to stressful circumstances during every work shift. In contrast, when the structure and content of mental health resources for HCWs are guided by the principles of PFA, support can be tailored to address where individuals are on the stress continuum in the present moment.

As the pandemic progresses, new geographical areas of the United States are experiencing overwhelming volumes of patients with COVID-19. Research from the SARS era identified 2 psychological phases of the mental health of HCWs during that epidemic: an initial shock and reaction phase when the number of SARS cases was exponentially growing, and a repair and reorientation phase as the epidemic ended (24). Distinct HCW stressors were observed during each phase, with anxiety predominating during the initial phase, and depression, somatic symptoms, and avoidance seen in the repair phase. With HCWs in the United States now spread across this continuum, we must be prepared to tailor mental health interventions for HCWs who are actively in crisis while also taking care of those who are coping during stages of recovery. The Stress Continuum Model is well adapted to addressing the temporal mental health needs of HCWs throughout the pandemic, because it is flexible and can adapt as the acuity of stressors changes. This approach is particularly important as future waves of COVID-19 are projected.

It is also critical that mental health strategies for HCWs during COVID-19 extend beyond individual-level interventions, as shown in Table 1. Previous research on the mental health of HCWs during SARS demonstrated that moral support, perceived adequacy of training and access to protective equipment, and clear communication from leadership were protective of adverse psychological outcomes (15). These findings have contributed to a focus on “organizational resilience,” a framework that recognizes that institutional, system-level interventions, including transparency, compassionate leadership, and access to practical resources such as hazard pay, temporary housing, and childcare, are in fact essential mental health interventions that can also mitigate the risk of acute and chronic stress (18,25,26).

Trainees are particularly at risk during the pandemic as frontline workers, and the emotions and daily adversities of medical students, residents, and fellows working in oncology should be validated during this crisis. The aforementioned PFA framework emphasizes the role of social support networks in enabling HCWs to cope with acute stressors, which may be a vitally important element for trainees (27). In the vein of organizational resilience, health-care leaders should maximize the control that trainees have over their educational environment when possible to mitigate the uncertainty precipitated by the pandemic. Efforts by trainees to collectively organize to advocate for their working conditions should also be championed by institutions, because trainees in particular are at risk for abuse during the pandemic which can threaten their mental health (28,29).

There is clearly a pressing need to support HCWs who are suffering, and we recognize the complexity of delivering mental health interventions while still learning how to optimally care for HCWs in the middle of an unfolding and unprecedented crisis. We must approach delivery of mental health with the same nuance, rigor, and care as the physical health of our patients with COVID-19. We know there are evidence-based individual and organizational interventions that can promote effective coping with traumatic stress. The challenge during the pandemic is to avoid pathologizing normal stress reactions and to provide appropriate supports to HCWs that are concordant with their needs on the stress continuum without inducing retraumatization. After all, primum non nocere*—*we must first do no harm.

## Funding

There are no funding sources to declare for this commentary.

## Notes

**Role of the funder:** Not applicable.

**Disclosures:** No author disclosures to declare for A.M., C.J.R., or S.B.

**Author contributions:** Conceptualization, A.M.; Investigation, A.M., C.J.R., and S.B.; Writing—Original Draft, A.M.; Writing—Review and Editing, C.J.R. and S.B.; Supervision, C.J.R. and S.B.

## Data Availability

Not applicable.
